# Expression of TPM1**κ**, a Novel Sarcomeric Isoform of the *TPM1* Gene, in Mouse Heart and Skeletal Muscle

**DOI:** 10.1155/2014/896068

**Published:** 2014-04-24

**Authors:** Syamalima Dube, Lauren Panebianco, Amr A. Matoq, Henry N. Chionuma, Christopher R. Denz, Bernard J. Poiesz, Dipak K. Dube

**Affiliations:** Department of Medicine, SUNY Upstate Medical University, 750 East Adams Street, Syracuse, NY 13210, USA

## Abstract

We have investigated the expression of TPM1**α** and TPM1**κ** in mouse striated muscles. TPM1**α** and TMP1**κ** were amplified from the cDNA of mouse heart by using conventional RT-PCR. We have cloned the PCR amplified DNA and determined the nucleotide sequences. Deduced amino acid sequences show that there are three amino acid changes in mouse exon 2a when compared with the human TPM1**κ**. However, the deduced amino acid sequences of human TPM1**α** and mouse TPM1**α** are identical. Conventional RT-PCR data as well as qRT-PCR data, calculating both absolute copy number and relative expression, revealed that the expression of TPM1**κ** is significantly lower compared to TPM1**α** in both mouse heart and skeletal muscle. It was also found that the expression level of TPM1**κ** transcripts in mouse heart is higher than it is in skeletal muscle. To the best of our knowledge, this is the first report of the expression of TPM1**κ** in mammalian skeletal muscle.

## 1. Introduction

Tropomyosins (TMs) are a family of highly conserved actin binding proteins which are expressed in all eukaryotes from yeast to humans. TMs play a critical role in the control of Ca^+2^-regulated thin filament function in striated muscle contraction. Except for zebrafish, in vertebrates, there are four known tropomyosin (TPM) genes (designated as* TPM1*,* TPM2*,* TPM3*, and* TPM4*) [1–6]. In zebrafish, six tropomyosin genes have been reported [7]. Each of the TPM genes generates a multitude of tissue and developmental specific isoforms via alternate splicing.* TPM1* is known to produce at least ten alternatively spliced transcript variants [8].

In mammals, the predominant striated muscle isoform is TPM1*α* containing exons 1a, 2b, 3, 4, 5, 6b, 7, 8, and 9a/b, which encode 284 amino acid residues. Our laboratory first reported another striated muscle isoform known as TPM1*κ* in axolotl heart [9] that contains exons 1a, 2a (instead of exon 2b), 3, 4, 5, 6b, 7, 8, and 9a/b. We also reported the expression of TPM1*κ* in axolotl skeletal muscle [8], human heart [10], and embryonic chicken heart [11]. In humans, TPM1*κ* is expressed in both fetal and adult hearts, whereas in chicken both TPM1*α* and TPM1*κ* are expressed only in embryonic heart [11]. Interestingly, transgenic mice that overexpressed human TPM1*κ*, in a cardiac-specific manner, were found to develop a dilated cardiomyopathy-like syndrome [12]. However, it was not clear whether TPM1*κ* is expressed in normal mouse hearts. Two possible reasons for observing some abnormalities in TG mouse hearts ectopically overexpressing human TPM1*κ* were that too much TPM1*κ* is toxic to the mouse cardiomyocyte or that human TPM1*κ* is variant from its mouse homologue and, for that reason alone, is toxic. Hence, we decided to explore TPM1*κ* expression in mouse hearts by RT-PCR and sequencing.

In this study, we report the expression of TPM1*κ* in mouse heart and skeletal muscles. To the best of our knowledge, this is the first report that TPM1*κ* is expressed in mammalian skeletal muscle. Two different methods were used to quantify the expression of TPM1*α* and TPM1*κ*. We determined the absolute copy number as well as relative expression compared to a reference gene (18S rRNA) in mouse heart and skeletal muscle. Unlike in humans [12], the expression level of TPM1*κ* transcripts in mouse heart is much lower compared to TPM1*α*. Further, there are three amino acid changes in mouse relative to human TPM1*κ*.

## 2. Materials and Methods

Mouse whole heart RNA was procured from Stratagen and ZyAgen; skeletal muscle RNA from BioChain and ZyAgen. Also, we received mouse whole heart and skeletal muscle RNA as a generous gift from Dr. David Wieczroek, University of Cincinnati, Cincinnati, OH. Each of these four sources of RNA came from a single adult mouse.

The quality of RNA was determined by capillary electrophoresis using 2100 Bioanalyzer, Agilent Technologies. The RIN (RNA integrity number) of the RNA samples ranged from 7.2 to 9, indicating that the RNAs we used were of good quality.

### 2.1. Preparation of cDNA for Conventional RT-PCR

0.5 *μ*g of RNA in a total volume of 40 *μ*L was used for preparing first strand cDNA synthesis with the SuperScript^R^ II (Life Technologies, Brand Island, NY) and oligodT primer following the manufacturer's specification. For each PCR amplification, 3 *μ*L of cDNA was used [13, 14]. Nucleotide sequences for the primer pairs for amplification of various mouse genes are given in Table 1.

### 2.2. Conventional RT-PCR, Cloning, and Sequencing of TPM1*κ* from Heart and Skeletal Muscle

**Figure 1:**
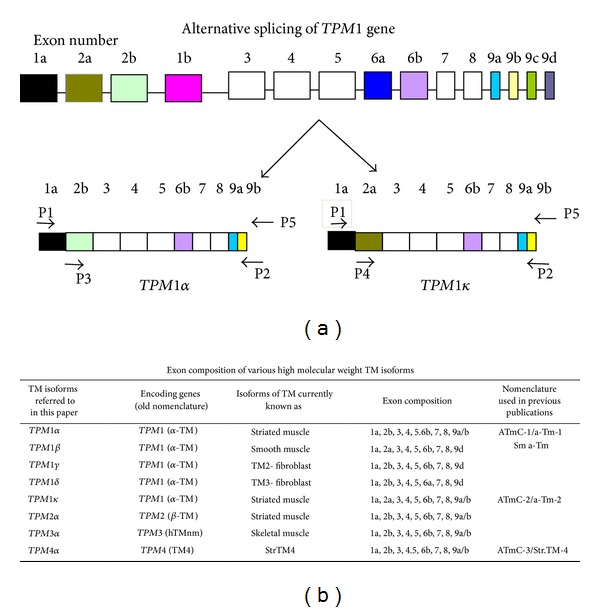
Alternate splicing of the* TPM1* gene and exon composition of various TM isoforms. (a) Splicing pattern of the TPM1 gene and the strategy for amplification of TPM1*α* and TPM1*κ*. Arrows indicate the primer pairs used for amplification of TPM1*α* and TPM1*κ* by RT-PCR and subsequent southern hybridization. The nucleotide sequences of various primer pairs and probes are listed in Table 1. (b) Exon composition of various TPM isoforms.

Figure 1 illustrates the pattern of alternative splicing of the two sarcomeric TPM1 isoforms. TPM1*α* contains exons 1a, 2b, 3, 4, 5, 6b, 7, 8, and 9a/b, whereas TPM1*κ* has all the same exons except it contains exon 2a instead of exon 2b. The figure also depicts the strategy for amplification of the two TPM1 isoforms. Primer pair P1(+)/P2(−) was used to amplify both TPM1*α* and TPM1*κ*. Amplification by the primer pairs P3(+)/P2(−) and P4(+)/P2(−) will yield TPM1*α* and TPM1*κ*, respectively. Subsequently, an isoform-specific [32P]-labeled probe was used for southern hybridization of TPM1*α* or TPM1*κ*. The sequences of various primer pairs as well as probes are shown in Table 1. We amplified TPM1*α* and TPM1*κ* by RT-PCR with cDNA from mouse heart muscle using the mus TPM1*α*/*κ* primer pair. The amplified DNA was run on a 1.5% agarose gel and the DNA band of ~850 bp was extracted using MinElute Gel Extraction kit of QIAGEN. The eluted DNA was ligated to T/A cloning vector (Invitrogen) following the manufacturer's specification as described earlier [10]. The ligation mix was used for transformation of competent one shot* E. coli* cells (Invitrogen) using the protocol supplied by Invitrogen. Appropriate hybridization positive colonies were picked up from the plates after filter hybridization with [32P]-labeled probe. Exon 2a specific probe was used for TPM1*κ* and exon 2b specific probe was used for TPM1*α*. Colonies were grown overnight with LB medium containing appropriate concentration of antibiotic and plasmid DNA was isolated using QIAprep Spin Miniprep kit (QIAGEN). Isolated plasmid DNA was sequenced at the Cornell University DNA sequencing facility (Figure 5).

### 2.3. cDNA for qRT-PCR

In order to exclude all other isoforms of the* TPM1* gene, cDNA was made with a negative primer from exon 9a (5′-CGCTCTCAACGATATGACTT-3′), which is common to both TPM1*α* and TPM1*κ* and is absent from other isoforms (Figure 1). 0.5 *μ*g of RNA in a total volume of 40 *μ*L was used for preparing the first strand cDNA synthesis with the SuperScript^R^ II (Life Technologies, Brand Island, NY) following the manufacturer's specification.

### 2.4. Real Time qRT-PCR

Real-time quantitative RT-PCR (qRT-PCR) analysis of cDNA template was performed using the LightCycler 480 real-time PCR system. Reactions were carried out in a 384-well plate using the LightCycler 480 SYBR Green I Master kit (Roche). Briefly, each well contained a total volume of 10 *μ*L, of which 2 *μ*L was cDNA template and 8 *μ*L was SYBR Green mix (5 *μ*L 1x SYBR Green Master mix, 2.8 *μ*L of PCR grade water, and 0.2 *μ*L of 10 *μ*M primer pair). Expression of TPM1 isoforms was determined using primer pairs for TPM1*α* and TPM1*κ*, (+)5′-TGGAAGATGAGCTGGTGTCAC-3′/(−)5′-TCAATGACTTTCATGCCTCT-3′, and (+)5′-CTCGAGGAGGACATCGCAGCG-3′/(−)5′-TCAATGACTTTCATGCCTCT-3′, respectively. 18S rRNA was assessed as an internal control with primer pair (+)5′-GTGGAGCGATTTGTCTGGTT-3′/(−)5′-CGCTGA GCCAGTCAGTGTAG-3′. Amplification in the absence of cDNA template was also evaluated to ensure lack of signal due to primer dimerization and extension or carryover. All samples and controls were performed in triplicate. End point melt curve analysis confirmed the presence of a single amplicon in each reaction well. A crossing threshold (CT) value was obtained, corresponding to the fractional number of amplification cycles where the PCR curve reaches a program-defined threshold amount of fluorescence. It is to be noted that qRT-PCR was performed separately with cDNAs prepared from RNA procured from three different sources as mentioned above.

Data were analyzed using both relative and absolute quantification methods. Relative quantification of qRT-PCR data was performed using the delta CT (sample CT minus 18S rRNA CT) and delta delta CT (sample delta CT minus comparator delta CT) methods [15, 16]. A comparative value was calculated using the formula *X* ^−delta  delta  CT^, where “*X*” equals the efficiency of TPM1*α* or TPM1*κ* primer pairs. This is similar to the 2^−delta  delta  CT^ method but corrects for the assumption that the reaction is occurring with 100% efficiency. Efficiencies (*E*) were determined using dilution series of TPM1*α* and TPM1*κ* plasmid clones with respective isoform-specific primers pairs. The LightCycler 480 software plotted the CT at each concentration against the logarithm of the fold dilution of the clone, generating a linear regression curve that calculated efficiency based on the formula *E* = 10^[−1/slope]^. Efficiencies were 79.05% and 88.42% for TPM1*α* and TPM1*κ*, respectively. In instances where TPM1*α* and TPM1*κ* were compared within the same cDNA sample, their efficiencies were averaged to equal 83.73%. Efficiency of 18S rRNA was determined by serial dilution of mouse cDNAs generated with exon 9a primer.

For determination of absolute copy number, optical density was taken of mouse TPM1*κ* and TPM1*α* TA clones separately using a spectrophotometer. The copy number per volume of clone in solution was determined using the equation number of copies = (ng of plasmid DNA × 6.02 × 10^23^)/(bp length of plasmid × 1 × 10^9^ × 650), which was simplified by Andrew Staroscik at the URI Genomics and Sequencing Center. A dilution series of each clone was done for 1 × 10^1^–1 × 10^4^ copies of template, which was used to create a standard curve after amplification [8, 14]. For better accuracy, each sample in dilution series was run in triplicate.

### 2.5. Statistical Analyses

The means, standard deviations, and comparative analyses of each data set for statistical significance were done using paired Student's *t*-test.

## 3. Results

### 3.1. Expression of TPM1*α* and TPM1*κ* in Mouse Heart and Skeletal Muscle

For expression analysis, we first employed conventional RT-PCR with cDNA made from total RNA from mouse heart and skeletal muscle. TPM1*κ* RNA was expressed in both mouse heart and skeletal muscle. To the best of our knowledge, this is the first report of the expression of TPM1*κ* in mammalian skeletal muscle, albeit at a significantly lower level compared to TPM1*α*.

**Figure 2:**
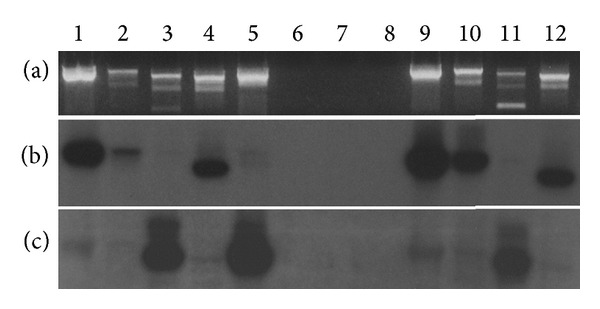
Expression analysis of TPM1*α* and TPM1*κ* RNA in mouse heart and skeletal muscle by RT-PCR. Plate (a) illustrates the ethidium bromide stain. Plate (b) illustrates hybridization with TPM1*α* probe. Plate (c) illustrates hybridization with TPM1*κ* probe. Lane 1: heart cDNA amplified with Mus TPM1*α*/*κ*-1 (+)/Mus UTR (−). Lane 2: heart cDNA amplified with Mus TPM1*α*/*κ*-1 (+)/Mus TPM1*α*/*κ*-2 (−). Lane 3: heart cDNA amplified with Mus TPM1*κ*-1 (+)/Mus TPM1*α*/*κ*-2 (−). Lane 4: heart cDNA amplified with TPM1*α* (+)/Mus TPM1*α*/*κ*-2 (−). Lane 5: heart cNDA amplified with Mus TPM1*κ*-1 (+)/Mus UTR (−). Lane 6: primer control for Mus TPM1*α*/*κ*-1 (+)/Mus UTR (−). Lane 7: primer control for Mus TPM1*α*/*κ*-1 (+)/Mus TPM1*α*/*κ*-2 (−). Lane 8: primer control for Mus TPM1*κ*-1 (+)/Mus TPM1*α*/*κ*-2 (−). Lane 9: skeletal cDNA amplified with Mus TPM1*α*/*κ*-1 (+)/Mus UTR (−). Lane 10: skeletal cDNA amplified with Mus TPM1*α*/*κ*-1 (+)/Mus TPM1*α*/*κ*-2 (−). Lane 11: skeletal cDNA amplified with Mus TPM1*κ*-1 (+)/Mus TPM1*α*/*κ*-2 (−). Lane 12: skeletal cDNA amplified with Mus TPM1*α*-1 (+)/Mus TPM1*α*/*κ*-2 (−).

Figure 2 depicts the results of conventional RT-PCR analysis of TPM1*α* and TPM1*κ* with cDNAs synthesized from mouse heart and skeletal muscle total RNA (provided by Dr. David Wieczroek), using a variety of generic as well as isoform specific primer pairs. Panel (a) in Figure 2 represents the ethidium stained PCR products run on a 1.5% agarose gel. Lanes 1 and 2 show the PCR products amplified from heart cDNA with two different primer pairs, which amplify both TPM1*α* and TPM1*κ*. Southern hybridization with TPM1*α*-specific probe demonstrated stronger signals in both lanes (lanes 1 and 2, panel (b)) compared to hybridization with TPM1*κ*-specific probe (lanes 1 and 2, panel (c)). The weaker signals in lanes 1 and 2 in panel (c) compared to panel (b) indicate much lower expression level of TPM1*κ* compared to TPM1*α*. Lanes 3 and 4 in panel (a) represent the ethidium staining of the PCR products when amplified with TPM1*κ* or TPM1*α*-specific primer pairs, respectively. Southern hybridization with TPM1*α*-specific probe shows a strong hybridization signal (lane 4, panel (b)) with the TPM1*α*-specific primer pair. A strong hybridization signal was also detected with TPM1*κ*-specific probe (lane 3, panel (c)) with the TPM1*κ*-specific primer pair. The results indicate that both TPM1*α* and TPM1*κ* RNA are expressed in mouse heart. This was further confirmed by amplification with another TPM1*κ*-specific primer pair and subsequent southern hybridization with TPM1*κ*-specific probe (lane 5, panels (a), (b), and (c)). All primer controls were negative (lanes 6, 7, and 8). Lanes 9 and 10 represent the PCR amplicons of mouse skeletal muscle cDNA with two sets of TPM1*α*/*κ* primer pairs. Ethidium bromide stained gels indicate a strong expression of TPM1 in skeletal muscle (panel (a)). Hybridization signal with TPM1*α*-specific probe (panel (b)) versus the TPM1*κ*-specific probe (panel (c)) indicates a much stronger expression of the former. However, amplification of skeletal muscle cDNA with TPM1*κ*-specific primer pair (lane 11 in panel (a)) and subsequent hybridization with TPM1*κ*-specific probe (lane 11 in panel (c)) validate the expression of TPM1*κ* in mouse skeletal muscle. Similarly, the expression of TPM1*α* in skeletal muscle was authenticated by the PCR amplification with TPM1*α*-specific primer pair (lane 12, panel (a)) and by subsequent hybridization with TPM1*α*-specific probe (lane 12, panel (b)). A much lower expression level of TPM1*κ* compared to TPM1*α* in mouse heart and skeletal muscle was further substantiated by qRT-PCR as described below. Similar conventional RT-PCR results were obtained from the RNA procured from Stratagen and Biochain (data not shown).

### 3.2. Determination of Copy Number of TPM1*α* and TPM1*κ* in Mouse Heart and Skeletal Muscles by qRT-PCR

**Figure 3:**
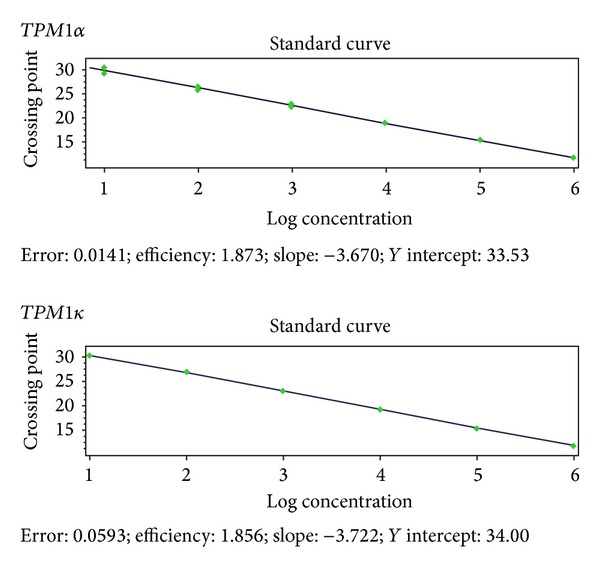
Quantitative RT-PCR of TPM1*α* and TPM1*κ*. Standard curve was derived from amplification of mouse TPM1*α* and TPM1*κ* plasmid clones after serial dilution. Copy number of TPM1*α* and TPM1*κ* was calculated per microgram of total RNA from mouse heart and skeletal muscle (Table 2).

Relative quantification of TPM1*α* and TPM1*κ* in mouse heart and skeletal muscles was performed on all sources of RNA. The standard curves for TPM1*α* and TPM1*κ* are shown in Figure 3. Our qRT-PCR data for copy number determination indicate that the expression of TPM1*κ* is much lower in both heart and skeletal muscle compared to TPM1*α* (Table 2). In relation to cardiac muscle, the data indicate that the level of expression of TPM1*α* transcripts is significantly higher and the level of TPM1*κ* is significantly lower in skeletal muscle (Tables 2 and 3).

### 3.3. Relative Expression of TPM1*α* and TPM1*κ*

**Figure 4:**
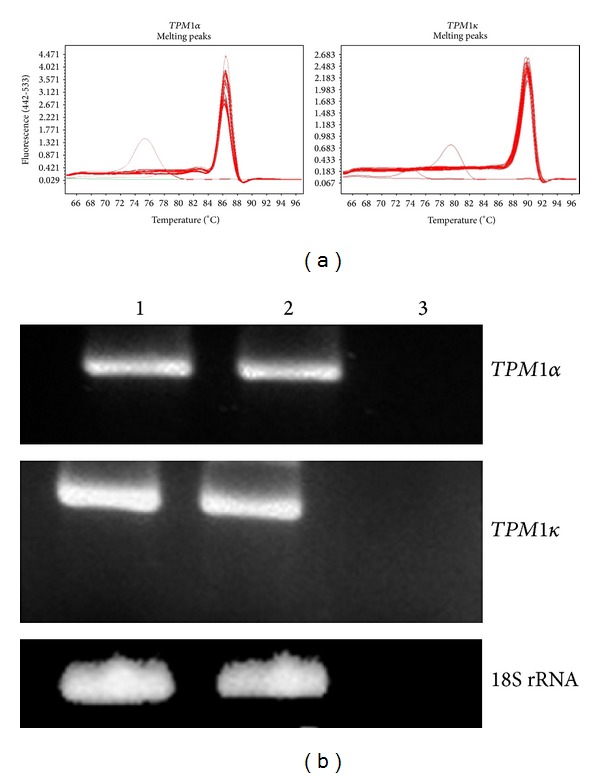
Melt curves of SYBER green PCR and the agarose gel electrophoresis of PCR products with ethidium bromide staining. (a) Melt curves for amplified TPM1*α* and TPM1*κ* DNA. The peaks melting at one different temperature specific for TPM1*α* or TPM1*κ* demonstrate specific amplification of just one product for TPM1*α* and TPM1*κ*. The multiple curves represent the products from multiple replicates of the RT-PCR assay. (b) Agarose gel of PCR products with ethidium bromide staining to demonstrate amplification of a single product for TPM1*α*, TPM1*κ*, and 18S rRNA amplification. Lane 1 is heart, lane 2 is skeletal muscle, and lane 3 is for the respective primer control.

As the absolute copy number determination revealed that the expression level of TPM1*κ* is significantly lower in heart and skeletal muscle, we analyzed our results by 2^∧^-(ddCT) method using 18S rRNA as the reference gene to determine the relative expression of TPM1*α* and TPM1*κ*. Melt curve data presented in Figure 4(a) show different unique melting temperatures for TPM1*α* (left) and TPM1*κ* (right). Also, agarose gel electrophoresis of the PCR amplified DNA for TPM1*α* or TPM1*κ* shows a single band for both of them (Figure 4(b)). Hence, each primer pair recognizes only TPM1*α* or TPM1*κ* amplified DNA.

The data in Table 4 validate our previous findings that expression of TPM1*κ* is significantly lower than TPM1*α* in mouse heart and skeletal muscle. The fold changes of TPM1*α*/TPM1*κ* are significantly higher in skeletal muscle suggesting the higher expression level of TPM1*κ* in heart as concluded from the absolute copy number data (Table 2). We analyzed our qRT-PCR data by 2^∧^-(ddCT). The results support our conclusion that TPM1*α* expression is much higher in mouse skeletal versus heart muscle; and the level of expression of TPM1*κ* is higher in mouse heart versus skeletal muscle (Table 5).

### 3.4. DNA Sequencing of TPM1*α* and TPM1*κ* cDNAs from Mouse Heart and Skeletal Muscle

**Figure 5:**
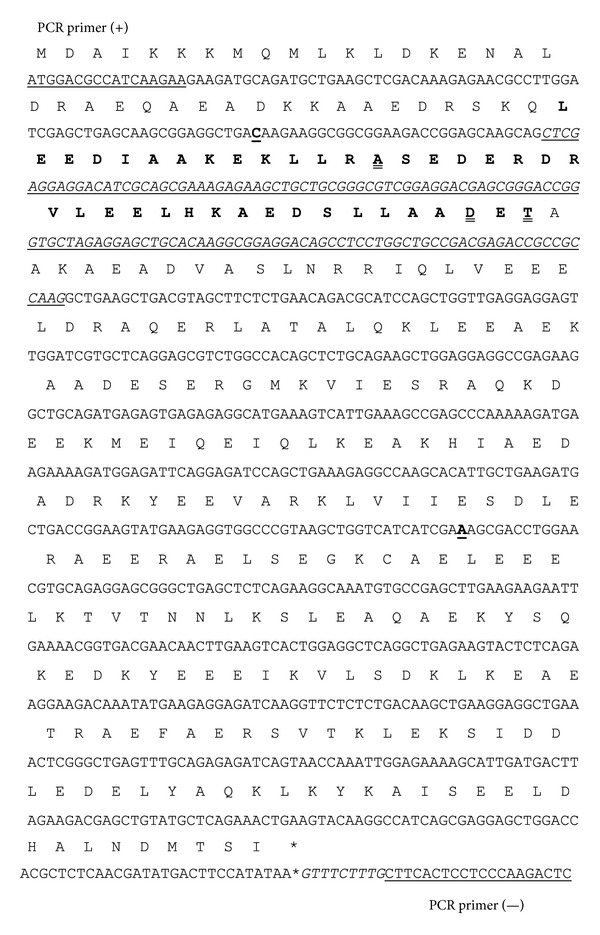
Nucleotide and deduced amino acid sequences of mouse TPM1*κ*. Nucleotide sequence exon 2a is underlined and italicized. The generic primer pair that amplifies both TPM1*α* and TPM1*κ* is underlined. Two nucleotides in TPM1*κ* in exons 1a and 4 were found to be different from the published sequence. TPM1*α* sequence is made bold and underlined. These changes do not alter the amino acid sequences. Deduced protein/amino acid sequences are on the top of the nucleotide sequences. Symbol of each amino acid residue is placed on the top of the third nucleotide of each codon. Amino acid residues in exon 2a that are different form human TPM1*κ* are underlined with double lines.

**Figure 6:**
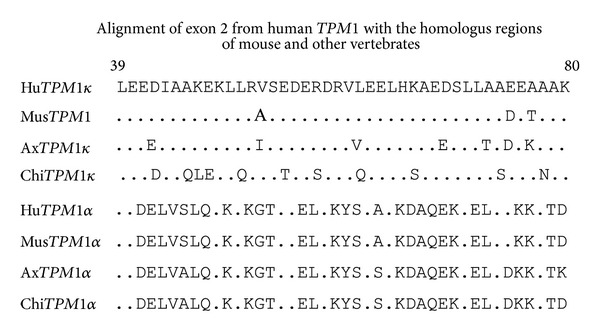
Alignment of the amino acid sequences of TPM1 exon 2a or 2b from various species. The top 4 sequences are 2a and the bottom four sequences are 2b. human (Hu), mouse (Mus), axolotl (ax), and chicken (Chi) sequences are shown. The underlined sequence in human TPM1*κ* was used to develop the antibody utilized to detect TPM1*κ* protein. The amino acid residue numbers are as shown. Conserved sequences are indicated by the (·).

cDNA sequencing confirmed the expression of both TPM1*α* and TPM1*κ* expression in mouse heart and skeletal muscle. Nucleotide and the deduced amino acid sequences of mouse TPM1*α* were identical with the published sequence (accession number NM_001164248.1) (data not shown). Also, the nucleotide sequences of mouse TPM1*α* and TPM1*κ* as depicted in Figure 5 are identical except for exon 2. As predicted, TPM1*α* contains exon 2b, but TPM1*κ* has exon 2a. The deduced amino acid sequences of mouse and human exon 2b of the* TPM1* gene are identical. The deduced amino acid sequences of exon 2a of mouse TPM1*κ* and the published exon 2a sequences in mouse TPM1*β* (smooth muscle TPM1 isoform) (NM_001164249.1) are also identical. However, some differences were noted when comparing the deduced amino acid sequences of mouse exon 2a with human TPM1 2a (Figure 6). Amino acid residues ^72^E and ^74^A in humans are replaced by ^72^D and ^74^T in mouse TPM1*κ*, respectively.

However, the most notable difference is at amino acid residue 52. In humans, it is valine, whereas in mouse it is alanine. This amino acid residue is in the middle of the 15-mer peptide (KEKLLR**V**SEDERDRV) that was used as the antigen for developing antibody against human TPM1*κ* [12]. This alteration may cause a lower affinity of this antibody towards mouse TPM1*κ* protein. In order to evaluate the expression of TPM1*κ* protein, in this study, we carried out western blot analysis with isolated myofibrils from mouse heart and skeletal muscle using the human TPM1*κ*-specific antibodies. The antibodies against human protein showed several nonspecific bands with mouse heart and skeletal muscle extracts. The western results are not convincing as to whether TPM1*κ* protein is expressed in mouse striated muscle.

## 4. Discussion

We first detected and sequenced TPM1*κ* from the heart of the Mexican axolotl [9]. Both the RNA and protein of this sarcomeric TM isoform is expressed in both heart and skeletal muscle in axolotl [8]. Subsequently, we reported the expression of TPM1*κ* transcripts and protein in human heart but not in skeletal muscle [10]. Also, TPM1*κ* along with TPM1*α* is expressed in embryonic chicken heart but not in skeletal muscle [11]. However, the transcripts of neither TM isoform could be detected in adult chicken heart and skeletal muscle [11]. Our present study clearly demonstrates that TPM1*κ* RNA is expressed in adult mouse heart and skeletal muscle. Unfortunately, because an anti-mouse TPM1*κ* antibody is not available, we cannot comment on protein expression. The levels of TPM1*α* and TPM1*κ* RNA in axolotl heart and skeletal muscle and human heart muscle are comparable. However, the level of expression of TPM1*κ* protein compared to TPM1*α* protein is very low. This suggests a differential translational efficiency for the transcripts of the two isoforms. Hence, we cannot speculate about levels of TPM1*κ* protein in murine striated muscles.

Interestingly, the expression of TPM1*κ* protein is increased in hearts in dilated cardiomyopathy (DCM) and heart failure human patients. However, we do not know whether this increase in TPM1*κ* is the cause or consequence of the cardiac disease [12]. The immunohistochemical analyses with TPM1*κ* antibody show that it is incorporated into axolotl cardiac and skeletal myofibrils and into human cardiac myofibrils [8]. Currently, the exact role TPM1*κ* protein plays in cardiac contractility is unclear. We have shown that transfection of antisense TPM1*κ* oligonucleotides into embryonic axolotl hearts inhibits the cardiac contractility in situ and also disarrays the cardiac myofibrils [17].

Herein, for expression analysis of TPM1*κ* transcripts we performed both conventional and real-time RT-PCR with total RNA from mouse heart and skeletal muscle using isoform specific primer pairs. RT-PCR data show the expression pattern of TPM1*α* and TPM1*κ* in mouse heart and skeletal muscle (Figure 4). We quantified the absolute copy number of TPM1*α* and TPM1*κ* expressed in heart and skeletal muscle. The results presented in Tables 2 and 3 show the higher copy number of TPM1*κ* expressed in mouse heart compared to skeletal muscle. Total copy number of TPM1*α* is higher in skeletal muscle. Hence, the difference in the ratio of copy numbers of TPM1*α* : TPM1*κ* is even more pronounced between the two tissues. The expression of TPM1*α* transcript is ~2- to 3-fold higher in skeletal muscle compared to heart. On the contrary, the expression of TPM1*κ* is ~3-fold higher in heart compared to mouse skeletal muscle. The real-time PCR (RT-PCR) results using copy number procedure is also reflected from the qRT-PCR data using 2^∧^-(dCT) method where we have used 18S rRNA as the reference gene (Table 4). We conclude that expression level of TPM1*κ* transcripts is higher in mouse heart compared to skeletal muscle. Further, the expression level of TPM1*κ* compared to TPM1*α* is significantly lower in both heart and skeletal muscle. Our analysis of qRT-PCR data by 2^∧^-(ddCT) method also supports the similar conclusions (Table 5). It is worth mentioning that TPM1*α* expressed per *μ*g of total RNA is considerably higher in skeletal muscle relative to heart muscle in axolotl as well [8]. We do not know whether this would be a consistent finding in all vertebrates. A higher expression level of TPM1*α* in skeletal muscle could be attributed to the higher requirement of TPM1*α* protein in skeletal muscle or due to the higher turnover of TPM1*α* RNA or protein in skeletal muscle. An altered dynamicity of tropomyosin in heart versus skeletal muscle may contribute to higher concentration of TPM1*α* mRNA observed in skeletal muscle. Wang et al. [18] compared the recovery rates of mature myofibrils in avian cardiomyocytes and skeletal muscle cells transfected with avian TPM1*α* or TPM1*κ* after photobleaching and noted the marked decreased rates of recoveries of both TPM1 isoforms in skeletal muscles compared with myofibrils in cardiomyocytes. The results suggest a lower dynamicity of tropomyosin in skeletal muscle. Hence, one can speculate that more transcripts of tropomyosin accumulate in the skeletal muscles cells or the transcriptional efficiency of the* TPM1* gene is significantly higher in skeletal muscle cells. Further studies are warranted for proposing a definitive explanation.

The deduced amino acid sequences show that three amino acid residues in exon 2a (Figure 6) are different from that of human sequences. In human TPM1*κ*, the 52nd amino acid residue is valine whereas it is alanine in mouse TPM1*κ*. This alteration may contribute to a lower affinity of the TPM1*κ* antibody [8, 12] that we used for western blot analysis to detect the expression of TPM1*κ* protein in mouse heart and skeletal muscle. There is such precedence in tropomyosin literature. An antibody, ARG1, targeted toward residues 214–226 (Tyr-Ser-Gln-Lys-Glu-Asp-Arg-Tyr-Glu-Glu-Glu-Ile-Lys) of human TPM1*α* can recognize human TPM1*α* but not Rat TPM1*α*. The amino acid sequence of human TPM1*α* differs from that of rat TPM1*α* by an Arg-to-Lys amino acid exchange in position 220 [19].

In addition, amino acid residues ^72^E and ^74^A in humans are replaced by ^72^D and ^74^T, respectively, in mouse TPM1*κ* (Figure 6). Overexpression of human TPM1*κ* protein in a cardiac-specific manner leads to the development of DCM in transgenic mice [12, 20]. It is to be noted that several missense mutations in exon 2b in TPM1*α* have been implicated in human DCM [21]. Olson et al. identified two mutations in exon 2b of the* TPM1* gene that altered two very conserved amino acids and also reversed the charges on the surface of tropomyosin [21]. The differences of the amino acid residues in exon 2a of human TPM1*κ* and mouse TPM1*κ* (Figure 6) do not alter the net charges. At this juncture, we are unsure whether the absolute overexpression of human TPM1*κ* and/or its altered exon 2a protein relative to the mouse sequence explains the DCM-like phenotype observed in transgenic mice [12]. Obviously, one should consider creating transgenic mice with mouse TPM1*κ* to better understand the physiological relevance of TPM1*κ* protein.

## Figures and Tables

**Table 1:** Primer pairs and probes used for amplification and detection of TPM1*α* and/or TPM1*κ*.

Name	Name as in Figure 1	Sequences
Mus TPM1*α*/*κ* − 1 (+)	P1	5′-ATGGACGCCATCAAGAAGAA-3′
Mus TPM1*α*/*κ* − 2 (−)	P2	5′-TTATATGGAAGTTATATCGTT-3′
Mus TPM1*α* − 1 (+)	P3	5′-CTCAAGGGCACTGAAGATGAA-3′
Mus TPM1*κ* − 1 (+)	P4	5′-CTCGAGGAGATCGCAGCG-3′
Mus TPM1*α* probe	P5	5′-CTGGAGCTGGCGGAGAAAAAGGCC-3′
Mus TPM1*κ* probe		5′-GGCGGAGGACAGCCTCCTGGCTGC-3′
Mus TPM1 UTR (−)		5′-GAGTCTTGGGAGGAGTGAAG-3′

**Table 2:** Copy number of TPM1*α* and TPM1*κ* in mouse heart and skeletal muscle.

Isoform	Absolute copy number/*μ*g of total RNA × 10^5^							
	Heart				Skeletal muscle			
	Expt 1	Expt 2	Expt 3	Ave	Expt 1	Expt 2	Expt 3	Ave
TPM1*α*	7.3 ± 0.06	5.5 ± 0.04	7.34 ± 0.03	6.71 ± 1.05	19.6 ± 0.4	23.4 ± 1.6	15.8 ± 1.4	19.6 ± 3.8
TPM1*κ*	0.013 ± 0.001	0.040 ± 0.004	0.02 ± 0.00	0.024 ± 0.010	0.004 ± 0.000	0.011 ± 0.002	0.001 ± 0.001	0.0055 ± 0.004
TPM1*α* : TPM1*κ*	561	137	367	355	4900	2127	9294	5440

(i) TPM1*α* heart : TPM1*α* skeletal muscle, *P* < 0.001.

(ii) TPM1*κ* heart : TPM1*κ* skeletal muscle, *P* < 0.001.

(iii) TPM1*α* heart : TPM1*κ* heart, *P* < 0.001.

(iv) TPM1*α* skeletal muscle : TPM1*κ* skeletal muscle, *P* < 0.001.

**Table 3:** Fold changes of TPM1*α* and TPM1*κ* in heart and skeletal muscle (by copy number).

Ratio of TPM1*α* in skeletal muscle : heart				Ratio of TPM1*κ* in heart : skeletal muscle			
Expt 1	Expt 2	Expt 3	Mean ± SD	Expt 1	Expt 2	Expt 3	Mean ± SD
2.68	4.25	2.15	3.0 ± 1.09	3.25	3.63	10.0	5.62 ± 3.79

**Table 4:** qRT-PCR for mouse heart and skeletal muscle.

Muscle type	Fold differences of TPM1*α*/TPM1*κ* (2^∧^-(dCT))*			Average
	Experiment 1	Experiment 2	Experiment 3	
Heart	650.05	843.08	879.13	790.75 ± 123.1
Skeletal muscle	5372.22	4374.45	6292.72	5346.22 ± 959.3

*TPM1*α* heart : TPM1*κ* heart, *P* < 0.001; TPM1*α* skeletal muscle : TPM1*κ* skeletal muscle *P* < 0.001. TPM1*α*/TPM1*κ* heart : TPM1*α*/TPM1*κ* skeletal muscle, *P* < 0.001.

**Table 5:** Fold changes of TPM1*α* and TPM1*κ* in heart and skeletal muscle (2^∧^-(ddCT)).

Ratio of TPM1*α* in skeletal muscle : heart*				Ratio of TPM1*κ* in heart : skeletal muscle*			
Expt 1	Expt 2	Expt 3	Average ± S.D.	Expt 1	Expt 2	Expt 3	Average ± S.D.
2.55	1.7	1.79	2.03 ± 0.66	3.38	5.88	10.04	6.41 ± 4.83

*TPM1*α* skeletal muscle : TPM1*α* heart, *P* < 0.001; TPM1*κ* heart : TPM1 skeletal muscle, *P* < 0.001.
